# Elevation of *Bombina variegata *peptide 8 in mice with collagen-induced arthritis

**DOI:** 10.1186/1471-2474-10-45

**Published:** 2009-04-30

**Authors:** Daitaro Kurosaka, Kentaro Noda, Ken Yoshida, Kazuhiro Furuya, Taro Ukichi, Eigo Takahashi, Maimi Yanagimachi, Isamu Kingetsu, Saburo Saito, Akio Yamada

**Affiliations:** 1 Division of Rheumatology, Department of Internal Medicine, Jikei University School of Medicine, Tokyo, Japan; 2 Department of Molecular Immunology, Institute of DNA Medicine, Jikei University School of Medicine, Tokyo, Japan

## Abstract

**Background:**

*Bombina variegate *peptide 8 (Bv8) is a small protein secreted by frog skin. Recently it has been shown to contribute to tumor angiogenesis in mouse model. The purpose of this study was to investigate Bv8 in mice with type II collagen-induced arthritis (CIA).

**Methods:**

We induced CIA in male DBA/1J mice. The severity of arthritis was evaluated based on an arthritis score. RNA was extracted from the joint, and examined for Bv8 mRNA expression by RT-PCR and real-time RT-PCR. Synovial tissue and bone marrow were immunohistochemically examined using anti-Bv8 antibody.

**Results:**

The level of Bv8 mRNA expression in the joint was below the detection limit in the control group, but was elevated in the CIA group, and was correlated with the arthritis score. In addition, an increase in Bv8-positive cells was observed in the synovium and bone marrow in the CIA group.

**Conclusion:**

Bv8 was elevated in the synovium and bone marrow of CIA mice, suggesting that Bv8 plays an important role in the pathogenesis of arthritis.

## Background

*Bombina variegata *peptide 8 (Bv8)/prokineticin-2 is a protein isolated from skin secretions of the frog *Bombina variegata *[1,2]. Endocrine gland-derived VEGF, also known as prokineticin-1, belongs to the same family as Bv8[3]. Bv8 has diverse functions, being involved in angiogenesis, gastrointestinal motility, neurogenesis, circadian rhythm regulation, hormone release, and the pain threshold [4,5].

Recently, Ferrara et al. reported an interesting study on angiogenesis: tumor-derived granulocyte colony-stimulating factor (G-CSF) mobilized bone marrow Bv8-positive cells to tumor sites, and these cells were CD11b+/Gr1+, promoting tumor angiogenesis via the mediation of Bv8[6]. On the other hand, angiogenesis is closely involved in the pathogenesis of type II collagen-induced arthritis (CIA), a model of rheumatoid arthritis [7,8]. In addition, an increase in CD11b+/Gr1+ cells was reported in the joints of CIA mice [9,10]. These observations suggest that Bv8 is involved in the pathogenesis of arthritis in CIA mice. Therefore, in this study, we investigated Bv8 in CIA mice.

## Methods

### Induction of arthritis in mice

All animal experiments were performed according to the Guidelines on Animal Experimentation of the Jikei University School of Medicine. Five-week-old male DBA/1J mice were purchased from Kyudo Ltd. (Fukuoka, Japan), and used after an acclimatization period of 1 week together as a group. Collagen-induced arthritis was induced as follows.[8] A 0.3% solution of bovine type II collagen(Cosmo Bio Co., Ltd., Tokyo, Japan) was emulsified in an equal volume of complete Freund's adjuvant (Difco Laboratories, Detroit, MI, USA). One hundred microlitres of this emulsion was injected intradermally into the dorsal root of the tail of 6-week-old male DBA/1J mice. This day was defined as day 0. Before arthritis onset, the mice received a booster injection of 0.3% bovine type II collagen solution emulsified in incomplete Freund's adjuvant on day 21, as described above for the primary injection. We used non-immunized mice as a control. Each experimental group consisted of five mice. mRNA analysis was performed twice, and the reproducibility was confirmed.

### Macroscopic evaluation of arthritis

The severity of arthritis was evaluated by the sum of the score for each wrist and ankle joint based on the following arthritis scale: 0, normal; 1, swelling of digits alone or mild swelling of wrist and ankle joints; 2, clear swelling of wrist and ankle joints; and 3, ankylosis or deformity of wrist and ankle joints.

### RNA extraction from joints

Mice were sacrificed under diethyl ether inhalation anesthesia on days 21, 24, 28, and 35 after the first immunization, and the fore- and hindlimbs were amputated 5 mm proximal to the wrist and ankle joints, respectively. RNA was extracted using ISOGEN (Nippon Gene Co., Ltd., Tokyo, Japan).

### RT-PCR

cDNA was synthesized using SuperScript™ II Reverse Transcriptase (Invitrogen, Tokyo, Japan). PCR was performed employing Gene Amp PCR System 9700 (Applied Biosystems, Foster City, CA, USA) using Ampli Taq Gold (Applied Biosystems, Foster City, CA, USA). Testis-derived RNA was used as a control. The primers used for Bv8 and GAPDH were as follows: Bv8, 5'-AGAGGAAGAAGGAGGTTC-3' and 5'-GAGTAAGGGTGTGTCTGTCT-3'; and GAPDH, 5'-TCACCATCTTCCAGGAGCG-3' and 5'-CTGCTTCACCACCTTCTTGA-3'.

### Real-time RT-PCR

cDNA was synthesized using a QuantiTect Reverse Transcription Kit (Qiagen K.K., Tokyo, Japan). Real-time PCR was performed in an ABI PRISM Sequence Detection System (Applied Biosystems, Foster City, CA, USA) using a QuantiTect SYBR Green PCR Kit (Qiagen K.K., Tokyo, Japan) and Quantitect Primer Assay (Mm Prok2 1 SG Quantitect Primer Assay, Mm Gapd 1 SG Quantitect Primer Assay and Mm vegfa 1 SG Quantitect Primer Assay; Qiagen K.K., Tokyo, Japan). The results were analyzed using the ΔΔCt method.

### Histological evaluation

On day 28, mice were anesthetized by an intraperitoneal injection of pentobarbital, followed by perfusion fixation with 4% paraformaldehyde. Subsequently, the fore- and hindlimbs were amputated 5 mm proximal to the wrist and ankle joints, respectively, and were immersion-fixed in 4% paraformaldehyde at 4°C for 2 days. The fixed limbs were delipidated with 100% ethanol at 4°C for 1 day, and then decalcified with EDTA at room temperature for 3 days. Thin sections were stained with H and E, and immunostained with anti-prokineticin 2 and anti Gr-1 antibodies. Thin paraffin sections were deparaffinized with xylene, and the xylene was removed with 100% ethanol. To activate the antigen, sections were incubated in 1 mM sodium citrate buffer at 98°C for 2 h. Endogenous peroxidase in sections was inactivated with a peroxidase-blocking reagent (Dako Cytomation Co., Ltd., Kyoto, Japan). The sections were washed with PBS, blocked with 10% normal rabbit serum, and incubated with the primary antibody, a 1:200 dilution of goat anti-mouse prokineticin 2 antibody (Santa Cruz Biotechnology, Inc., Santa Cruz, CA, USA), at 4°C overnight, and washed with PBS. Subsequently, the sections were reacted with Simple Stain MAX-PO(G) (Nichirei Bioscience Inc., Tokyo, Japan) as the secondary antibody at room temperature for 30 min, and washed with PBS. The color was developed by the addition of Simple Stain DAB Solution (Nichirei Bioscience Inc., Tokyo, Japan) for 10 min at room temperature. The sections were finally counterstained with hematoxylin. For Gr-1 staining, the sections were incubated with primary antibody, a 1:100 dulution of rat anti-mouse Gr-1 antibody (Becton, Dickinson and Co., Tokyo, Japan), at 4°C overnight, and reacted with biotin-conjugated rabbit anti-rat IgG antibody (Dako Cytomation Co., Ltd., Kyoto, Japan) and ALP-labeled streptavidin (Nichirei Bioscience Inc., Tokyo, Japan). The color was developed by the addition of Vector Blue(Vector Laboratories, Inc., CA, USA)for 45 min at room temperature.

### Statistical analysis

Data obtained by real-time PCR were analyzed using the Mann-Whitney U-test. A p-value of less than 0.05 was considered significant.

## Results

**Figure 1:**
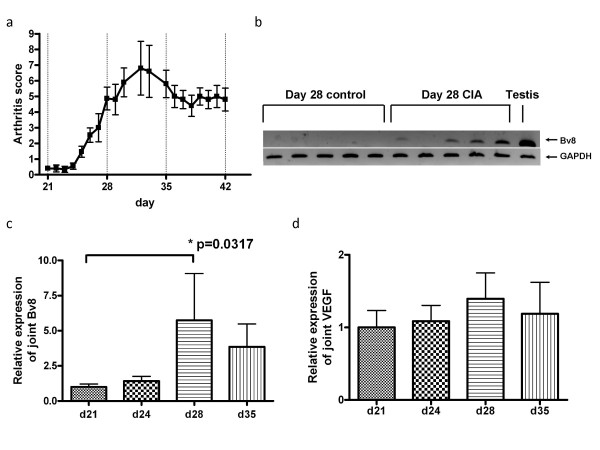
**Correlation between the arthritis score and Bv8 mRNA expression**. One group consisted of 5 mice. b shows the results of analysis by RT-PCR. c and d show the results of analysis by real-time PCR. The relative expression of Bv8 and VEGF mRNA was standardized to that of GAPDH mRNA. The results are expressed relative to day 21 CIA mice, arbitrarily assigned a value of 1.0. a. Changes in the arthritis score. b. Comparison of Bv8 mRNA expression in joint tissue on day 28 between the CIA and control groups. c. Bv8 mRNA expression in arthritic joints on days 21, 24, 28, and 35. d. VEGF mRNA expression in arthritic joints on days 21, 24, 28, and 35.

**Figure 2:**
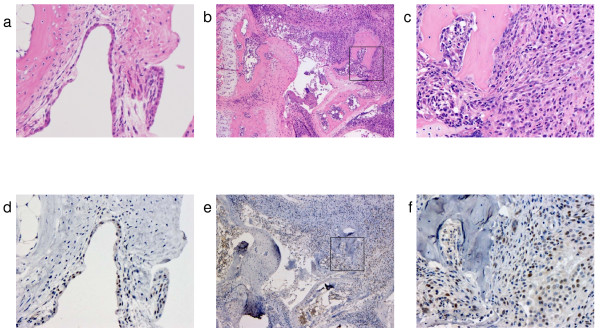
**Histopathological examination of the synovium**. Bv8 in Figs. d, e, and f was stained brown. a. HE-staining of the synovium in the control group (×200). b. HE-staining of the synovium in the CIA group (×50). c. HE-staining of the synovium in the CIA group (×200). The area in the box in Fig. b is enlarged. d. Immunohistochemical staining of the synovium with anti-Bv8 antibody in the control group (×200). e. Immunohistochemical staining of the synovium with anti-Bv8 antibody in the CIA group (×50). f. Immunohistochemical staining of the synovium with anti-Bv8 antibody in the CIA group (×200). The area in the box in Fig. e is enlarged.

**Figure 3:**
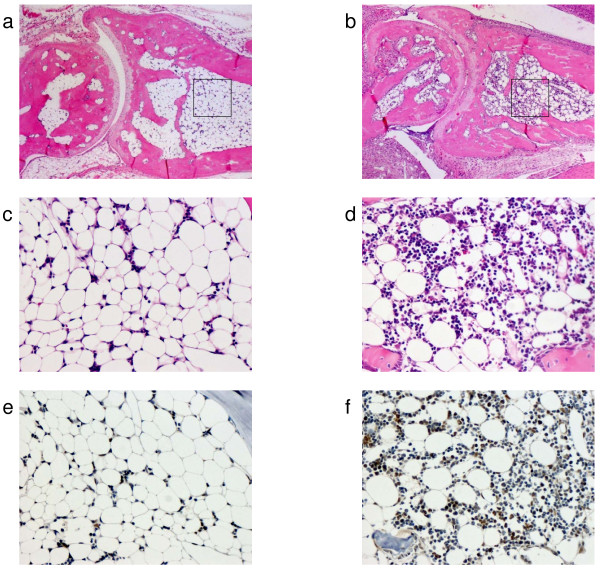
**Histopathological examination of bone marrow in the distal tibia**. In Figs. e and f, Bv8 was stained brown. a. HE-staining of bone marrow in the control group (×50). b. HE-staining of bone marrow in the CIA group (×50). c. HE-staining of bone marrow in the control group (×200). The area in the box in Fig. a is enlarged. d. HE-staining of bone marrow in the CIA group (×200). The area in the box in Fig. b is enlarged. e. Immunohistochemical staining of bone marrow with anti-Bv8 antibody in the control group (×200). f. Immunohistochemical staining of bone marrow with anti-Bv8 antibody in the CIA group (×200).

**Figure 4:**
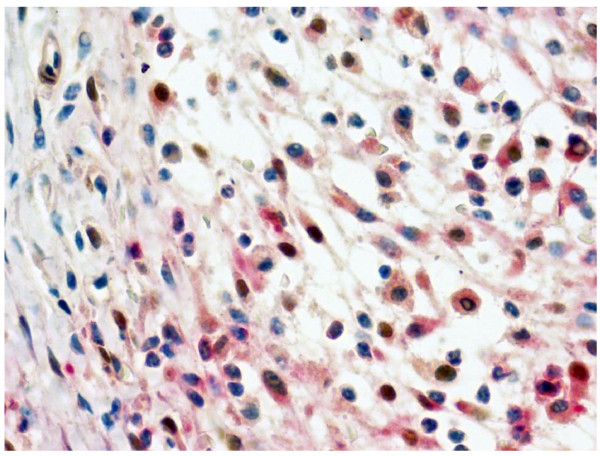
**Immunohistochemical staining of the synovium with anti-Bv8 and anti-Gr-1 antibodies in the CIA group (×400)**. Bv8 and Gr-1 were stained brown and red, respectively.

Mice developed CIA 2–3 days after the booster injection (on day 21). The severity of arthritis peaked on approximately day 30 (Fig. 1a). RNA was extracted from the joint on days 21, 24, 28, and 35, and was analyzed for Bv8 mRNA expression. On day 28, Bv8 mRNA expression was detectable by RT-PCR in 4 of the 5 CIA mice, but was below the detection limit in all control mice (Fig. 1b). Similar results were obtained in the CIA and control mice sacrificed on day 35 (data not shown). It is of note that Bv8 mRNA expression could not be detected in the RNA of CIA mice with minimal arthritis. Next, changes in Bv8 mRNA expression were analyzed by real-time RT-PCR (Fig. 1c). The level of Bv8 expression increased rapidly from day 28, and tended to decrease gradually thereafter. The time course of Bv8 mRNA expression roughly paralleled the arthritis score. Mann-Whitney U-test analysis showed significant differences in the Bv8 mRNA expression level between the mice sacrificed on days 21 and 28. The expression of mRNA for VEGF, an angiogenesis-promoting factor, was similarly analyzed. The level of VEGF expression was slightly higher on days 28 and 35 than on day 21 (Fig. 1d). The relative VEGF mRNA expression level was 1.39, as compared to 5.75 for Bv8, indicating that the rate of increase in Bv8 was higher than that in VEGF in arthritis. Next, to analyze Bv8 expression at the protein level, we examined synovial tissue and bone marrow immunohistochemically using anti-Bv8 antibody(Figs. 2, 3). Bv8-positive cells were observed in the superficial layer of the normal synovium (Fig. 2d), but were clearly increased in the arthritic pannus (Fig. 2e, f). Morphologically, most Bv8-positive cells were macrophage-like, although some of them could not be clearly identified. In addition, Bv8-positive cells were observed in the normal bone marrow of the distal tibia as well(Fig. 3e), but were markedly increased in the bone marrow of CIA mice(Fig. 3f). Morphologically, Bv8-positive cells were predominantly myeloid-lineage cells(Fig. 3c, d). To characterize Bv8-positive cells in more detail, arthritic joints were double-stained with anti-Bv8 and anti-Gr-1 antibodies. Most Bv8-positive cells observed in arthritic synovia were also positive for Gr-1 (Fig. 4).

## Discussion

The development of arthritis was associated with elevated Bv8 mRNA expression in the arthritic joint of CIA mice. Histopathologically, Bv8-positive cells were increased in the synovium and bone marrow. These results suggest that Bv8 is involved in the pathogenesis of arthritis. Further, the time course of changes in the expression of mRNA for VEGF, a potent angiogenesis-promoting factor, paralleled that in the expression of Bv8 mRNA. VEGF is known to play an important role in angiogenesis in inflamed synovia. The similar behavior of both factors in arthritis suggests the possible involvement of Bv8 as well as VEGF in angiogenesis in inflamed synovia. In this study, the rate of increase in Bv8 was higher than that in VEGF in arthritis. In normal joints, Bv8 is expressed very weakly, but VEGF expression is higher, which may explain the higher rate of increase in Bv8. In addition to being involved in angiogenesis, Bv8 has diverse functions, including circadian rhythm regulation[11]. Patients with rheumatoid arthritis exhibit an abnormal circadian rhythm[12], which has also been reported in a rat model of arthritis [13,14]. Therefore, Bv8 may be involved in arthritis pathogenesis. Ferrara et al. reported Bv8-expressing CD11b+/Gr1+ cells [6.15]. On the other hand, these cells have been shown to be increased in arthritic joints and peripheral tissue in arthritis models [9,10]. These observations strongly suggested that the Bv8-positive cells in the present study corresponded to CD11b+/Gr1+ cells. Therefore, we attempted to clarify this point, and found that the Bv8-positve cells in the synovium were positively immunostained with anti-Gr1 antibody; however, we could not detect the presence of CD11b in them. Since the tissue sections under study contained osseous and cartilaginous tissue requiring prolonged decalcification, it was necessary to prevent antigen denaturation by employing a fixation procedure. Only anti-CD11b antibody for frozen sections was available, which may have affected the analysis of CD11b expression. This point requires further study.

G-CSF has been reported to increase Bv8 expression in bone marrow CD11b+/Gr1+ cells, and to be important when Bv8-positive cells are mobilized to tumor tissue [6.16]. G-CSF is closely involved in the pathogenesis of arthritis as well[17]. Therefore, bone marrow CD11b+/Gr1+ cells may be mobilized to arthritic synovial tissue. On the other hand, some Bv8-positive cells were noted in normal synovial tissue. Further studies are necessary to determine what percentage of the Bv8-positive cells that were increased in arthritic synovial tissue were derived from the bone marrow.

## Conclusion

In this study, we demonstrated increased Bv8 expression in the synovium and bone marrow of CIA mice. Further studies are needed to clarify how theses changes are involved in the pathogenesis of arthritis.

## Competing interests

The authors declare that they have no competing interests.

## Authors' contributions

DK and KN designed the study and drafted the manuscript, KN and KF performed the experimental work and the statistical analysis. KY, TU, ET, MY, IK, SS and AY participated in study design and approved the final manuscript.

## Pre-publication history

The pre-publication history for this paper can be accessed here:


